# Automated Retinal Layer Segmentation Using Spectral Domain Optical Coherence Tomography: Evaluation of Inter-Session Repeatability and Agreement between Devices

**DOI:** 10.1371/journal.pone.0162001

**Published:** 2016-09-02

**Authors:** Louise Terry, Nicola Cassels, Kelly Lu, Jennifer H. Acton, Tom H. Margrain, Rachel V. North, James Fergusson, Nick White, Ashley Wood

**Affiliations:** 1 School of Optometry and Vision Sciences, Cardiff University, Cardiff, United Kingdom; 2 Vision Science Bioimaging Labs, Cardiff University, Cardiff, United Kingdom; Simon Fraser University, CANADA

## Abstract

Retinal and intra-retinal layer thicknesses are routinely generated from optical coherence tomography (OCT) images, but on-board software capabilities and image scaling assumptions are not consistent across devices. This study evaluates the device-independent Iowa Reference Algorithms (Iowa Institute for Biomedical Imaging) for automated intra-retinal layer segmentation and image scaling for three OCT systems. Healthy participants (n = 25) underwent macular volume scans using a Cirrus HD-OCT (Zeiss), 3D-OCT 1000 (Topcon), and a non-commercial long-wavelength (1040nm) OCT on two occasions. Mean thickness of 10 intra-retinal layers was measured in three ETDRS subfields (fovea, inner ring and outer ring) using the Iowa Reference Algorithms. Where available, total retinal thicknesses were measured using on-board software. Measured axial eye length (AEL)-dependent scaling was used throughout, with a comparison made to the system-specific fixed-AEL scaling. Inter-session repeatability and agreement between OCT systems and segmentation methods was assessed. Inter-session coefficient of repeatability (CoR) for the foveal subfield total retinal thickness was 3.43μm, 4.76μm, and 5.98μm for the Zeiss, Topcon, and long-wavelength images respectively. For the commercial software, CoR was 4.63μm (Zeiss) and 7.63μm (Topcon). The Iowa Reference Algorithms demonstrated higher repeatability than the on-board software and, in addition, reliably segmented all 10 intra-retinal layers. With fixed-AEL scaling, the algorithm produced significantly different thickness values for the three OCT devices (P<0.05), with these discrepancies generally characterized by an overall offset (bias) and correlations with axial eye length for the foveal subfield and outer ring (P<0.05). This correlation was reduced to an insignificant level in all cases when AEL-dependent scaling was used. Overall, the Iowa Reference Algorithms are viable for clinical and research use in healthy eyes imaged with these devices, however ocular biometry is required for accurate quantification of OCT images.

## Introduction

Optical coherence tomography (SD-OCT) is an essential imaging tool for the diagnosis and monitoring of retinal diseases such as age-related macular degeneration (AMD) [1,2] and diabetic macular oedema [3,4]. This non-invasive technique allows clinicians to produce three-dimensional (3-D) images of intraocular structures *in vivo*. In addition to subjective qualitative assessment, images can be analyzed objectively, providing quantitative measurements including retinal thickness. Such metrics are commonly used clinically and as outcome measures in research, for example in clinical trials [5,6]. Quantitative analysis of OCT images has become increasingly sophisticated [7,8] as image quality and software capabilities improve.

Whilst manual caliper tools and hand segmentation (hand tracing of intra-retinal layer boundaries) can be simple to perform, it is time consuming (particularly when implemented in 3-D scans) and subject to significant inter-observer variation [9]. These methods are therefore not feasible for use clinically or in large, multi-center clinical trials.

Commercial OCT devices are generally supplied with on-board segmentation software, designed to generate fast, reliable values for interpretation by clinicians. This software has historically been limited to total retinal thickness. Additionally, the definition of the retinal boundaries varies between manufacturers, with different reflective bands, as seen in OCT images, being chosen to represent the posterior retinal margin. This makes quantitative retinal thickness comparisons between commercial devices difficult [10]. Some instruments, such as the Topcon DRI OCT-1 Atlantis (Topcon Corp, Tokyo, Japan) and the latest Spectralis OCT (Heidelberg Engineering, Heidelberg, Germany), are now supplied with software that is capable of segmenting a number of intra-retinal layers. Furthermore, the commercial software is almost always limited to use with images captured by the parent device, and cannot be applied to images from other OCT devices. The diversity of segmentation methods and normative values confound comparisons between commercial systems [11–13].

OCTSeg is a module of OCT Explorer, itself part of the Iowa Reference Algorithms (Retinal Image Analysis Lab, Iowa Institute for Biomedical Imaging, Iowa City, IA). It is a publicly available, device-independent, graph theory-based tool for segmentation of 10 retinal layers in volumetric OCT images [7,14,15]. It produces retinal thickness values comparable to manual measurements of OCT images by retinal specialists [14,16], and to analysis by the Heyex software (Heidelberg Engineering) of images from participants with diabetic macular oedema [17]. Unlike the majority of commercial software, it can be applied to images from all widely-available clinical OCT devices, allowing direct comparison of images from multiple devices.

Since the segmentation software may be used with images from several different OCT instruments, it is important to establish agreement between devices. The majority of current devices utilize broad-band light sources with centre wavelengths (λ_c_) of ~850nm, but longer wavelength OCT (λ_c_ ~1040nm) has recently become commercially available. The algorithm may perform differently on these images, due to differences in reflectivity of retinal layer boundaries at these wavelengths. We used a non-commercial long-wavelength OCT (λ_c_ ~1040nm) in our evaluation of the algorithm.

Based on the work of Littmann and others [18,19], the transverse size of any retinal feature can be calculated using the appropriate ocular biometry and instrument meta-data. A reasonably accurate lateral scaling (converting feature sizes from pixels to microns) of any OCT image can be determined using (i) an estimate of the principal plane to retina distance of the eye (axial eye length (AEL) - 1.8mm), (ii) the angle of the OCT scan in air and (iii) an estimate of the bulk ocular refractive index. The on-board software of the two commercial instruments used in this study provide only a single lateral scaling value for all patients regardless of AEL, resulting in an error in the reported size of the image which scales with AEL. This error manifests as a discrepancy between the fixed size ETDRS grid and the AEL-dependent image size. Therefore, the position of retinal layer measurements and hence the regional thickness values become AEL-dependent; an important consideration when comparing different OCT instruments. By converting all our image files to a compatible TIFF format we were able to use the ability of OCTExplorer to accept an independently calculated, AEL-dependent lateral scaling for images from all devices used.

To the authors’ knowledge, inter-session repeatability of the Iowa Reference Algorithms has not been assessed to date. Given the potential clinical and research utility of quantitative intra-retinal layer analysis [20–22], investigation of this feature of the algorithm is a key step in validating the sensitivity of this tool to detect retinal changes. Furthermore, to our knowledge, there has been no formal comparison between retinal thickness measurements produced by the Iowa algorithm and the on-board segmentation algorithms of many commonly available commercial devices.

The aims of the present study are to evaluate the use of Iowa Reference Algorithms as a means of generating repeatable intra-retinal layer thickness values from images of healthy eyes, captured using two commercial SD-OCT devices (λ_c_ ~850nm) and one non-commercial long-wavelength (λ_c_ ~1040nm) device. Secondary objectives are to assess agreement between the algorithm and commercial, device-dependent software, as well as inter-device agreement.

## Materials and Methods

### Participants

Healthy participants (n = 25) were recruited from staff, students and volunteers at the School of Optometry and Vision Sciences, Cardiff University. All participants had a corrected visual acuity of 0.0 logMAR (20/20) or better using a high contrast Early Treatment of Diabetic Retinopathy Study (ETDRS) chart and a mean refractive error of ≤ ±6.00 diopters in the test eye. Approval for this study was obtained from the South East Wales NHS Research Ethics Committee. All experimental procedures adhered to the tenets of the Declaration of Helsinki, and written informed consent was obtained before data collection commenced.

Participants with disease affecting retinal function were excluded, including diabetes, glaucoma, and significant media opacities (Lens Opacities Classification System III grade 3 or more for any criteria [23]). These were identified using a medical history questionnaire, slit lamp examination and fundus photography. Those with narrow iridocorneal angles (grade 1 or less assessed by Van Herick) or intraocular pressure over 21mmHg were also excluded, as were those taking medication known to affect retinal function.

One eye was selected as the test eye for each participant; this was the eye with the better visual acuity (or lower refractive error if one eye was outside the ±6.00D range). One drop of Tropicamide 0.5% was instilled into the test eye of each participant prior to imaging. Following sufficient pupil dilation, fundus photographs were obtained to ensure participants did not have retinal disease. AEL (cornea to retinal pigment epithelium (RPE)) was measured using optical biometry (IOL Master, Carl Zeiss, Jena, Germany) to enable accurate lateral scaling calculations for all OCT images.

### OCT Imaging

OCT images were obtained from all participants using a 3D-OCT 1000 (Topcon Corp, Tokyo, Japan), Cirrus HD-OCT (Carl Zeiss Meditec, Inc., Dublin, CA), and a non-commercial long-wavelength (λ_c_ 1040nm) SD-OCT [24–26]. Images from the latter were obtained using less than the maximum permissible corneal exposure for unlimited duration at this wavelength (less than 5mW/cm^2^ averaged over a 7mm pupil) [27].

Volume scans centered on the fovea were acquired using each device. For comparison, all images had a scan angle of 20° x 20°, comprising 512 x 128 A-scans. Following dilation, all images were obtained by a single trained operator (LT). A second session was conducted within 1 month, using the same protocol and scheduled for the same time of day as the previous session (± 30 minutes).

### Data Analysis

Long-wavelength OCT images were exported to Fiji (Rasband; National Institute of Health, USA) [28] and underwent stack registration, using the Fiji plugin StackReg [29], to remove eye movement artefacts. Images were then exported to OCTExplorer 3.5. Images from the two commercial instruments were directly imported into the Iowa software, with no alteration when using the instrument-supplied scaling, or via conversion to a standard TIFF format (via Fiji) in order to apply an AEL-dependent lateral scaling.

**Fig 1:**
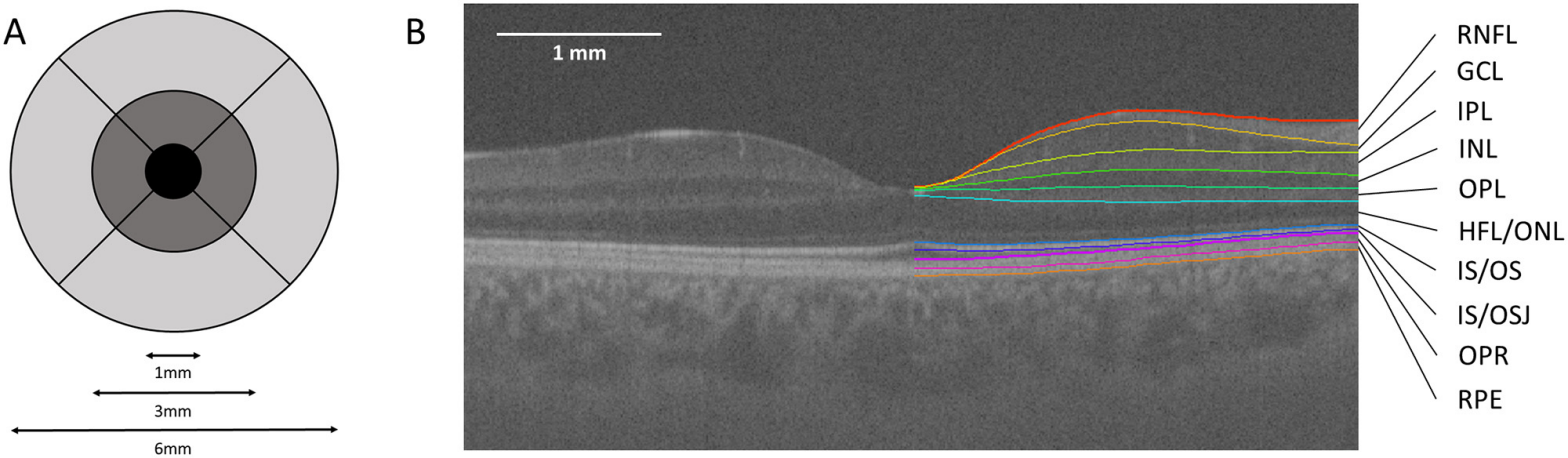
ETDRS grid and example 10 intra-retinal layer segmentation. (A) Standard ETDRS grid showing the foveal subfield (black). The inner ring is an average of the four parafoveal subfields (dark grey) and the outer ring of the four perifoveal subfields (light grey). (B) Screenshot of 10 layer (11 boundary) segmentation of a long-wavelength OCT image, produced by the Iowa Reference Algorithms. The left half of the image shows the image prior to segmentation. Layers 1–10 (top to bottom; as defined by the software): retinal nerve fiber layer (RNFL); ganglion cell layer (GCL); inner plexiform layer (IPL); inner nuclear layer (INL); outer plexiform layer (OPL); outer plexiform layer-Henle fiber layer to boundary of myoid and ellipsoid of inner segments (OPL-HFL ~ BMEIS); photoreceptor inner/outer segments (IS/OS); inner/outer segment junction to inner boundary of outer segment photoreceptor/retinal pigment epithelium complex (IS/OSJ ~ IB_RPE); outer segment photoreceptor/retinal pigment epithelium complex (OPR); retinal pigment epithelium (RPE).

Automated retinal layer segmentation using the Iowa Reference Algorithms was performed on all images. Mean retinal thickness values of 10 retinal layers were obtained on all images for the foveal subfield and the inner and outer rings of a standard ETDRS grid (shown in Fig 1). Total retinal thickness was also calculated, as the distance from the most anterior hyper-reflective line (corresponding to the inner limiting membrane; ILM) to the posterior of the outermost hyper-reflective line (corresponding to the outer boundary of RPE).

For all comparisons with the commercial software, the posterior retinal boundary used by the commercial segmentation was adopted in the Iowa Reference Algorithm segmentation (inner boundary of outer segment photoreceptor/RPE complex, and inner boundary of RPE for Topcon and Zeiss respectively; see Fig 1). These parameters will be referred to in this paper as ‘equivalent retinal thicknesses’.

For images from the two commercial instruments, meta-data supplied by the manufacturer were used in determining lateral pixel size for analyses using the on-board software to show the importance of correct scaling. In all other cases, lateral scaling was obtained from the scan angle, measured AEL and an assumed bulk ocular refractive index of 1.336. Axial scaling of all images was obtained from the supplied instrument meta-data. This included a group refractive index for retinal tissue of 1.4, 1.38 and 1.36 for the long-wavelength, Topcon and Zeiss instruments respectively.

Coefficients of repeatability (CoR), a measure of repeatability familiar to clinicians [30], were used to assess the inter-session variations in retinal thickness values produced by both the Iowa Reference Algorithms and on-board software of each device (versions 3.51.003.04 and 7.0.1.290 for the Topcon and Zeiss instruments respectively). CoR was calculated as 1.96 times the standard deviation (SD) of the differences between sessions [31], and is also expressed as a percentage of the mean retinal layer thickness. Bland-Altman plots [31] were used to assess agreement between these segmentation methods, and for inter-device comparisons. The bias (mean difference) and 95% limits of agreement (LoA) were calculated for each comparison, and Friedman’s two way ANOVA was used to identify statistically significance differences. In these comparisons, data points outside ±3 SD of the difference were defined as significant outliers (by Grubbs’ test [32] for n = 25) and were excluded from the analysis. These were classified as failings of the segmentation algorithm.

The relationship between retinal thickness discrepancies using the commercial instrument scaling and the AEL-correct scaling for our long wavelength images vs measured AEL was evaluated using Spearman’s rank correlation.

## Results

Twenty-five eyes from 25 healthy participants were included in the study. The age range of the participants was 20 to 62 years (mean ± SD, 34.9 ± 13.5 years). Sixteen participants were female (64%). The mean AEL and refractive error (mean sphere) were 23.7 ± 1.3 mm (range 21.6 to 26.6) and -0.58 ± 1.93 diopters (range -4.50 to +3.00) respectively. The maximum cylindrical power was 3.00 diopters.

### Lateral scaling for AEL

OCTSeg reported automated segmentation of 10 intra-retinal layers for all images. Mean values across all eyes for each retinal layer are shown in Table 1. These values underwent AEL-corrected lateral scaling as described above. The percentage difference of these corrected values from the fixed scaling values (by OCTSeg) ranged from -15% to +26% (corresponding to Topcon and Zeiss GCL layer respectively, both in the foveal subfield). Mean total retinal thickness measurements produced by both segmentation methods for the three devices can be seen in Table 2. These values underwent fixed-AEL scaling to allow comparison to the commercial software. In this case, correcting for AEL yielded mean differences of less than 2% in all cases when compared to the fixed-AEL scaling (by OCTSeg).

**Table 1:** Mean thickness of 10 intra-retinal layers. Thickness values (mean ± SD; μm) produced by segmentation of images at session 1 using the Iowa Reference Algorithms.

Layer	Fovea			Inner ring			Outer ring		
	Topcon	Zeiss	1040nm	Topcon	Zeiss	1040nm	Topcon	Zeiss	1040nm
**1. RNFL**	7.0 ± 2.3	5.9 ± 2.9	5.2 ± 2.2	25.5 ± 2.3	25.2 ± 2.3	24.2 ± 2.4	39.3 ± 7.0	40.9 ± 5.9	39.3 ± 5.0
**2. GCL**	17.0 ± 6.3	13.4 ± 5.4	17.2 ± 5.1	48.5 ± 5.9	52.4 ± 5.7	50.5 ± 6.2	24.7 ± 3.0	27.2 ± 3.1	29.3 ± 3.8
**3. IPL**	29.3 ± 2.9	27.5 ± 4.1	24.4 ± 4.2	42.5 ± 4.2	40.7 ± 3.5	39.9 ± 4.2	35.3 ± 2.8	37.3 ± 2.8	36.0 ± 3.8
**4. INL**	17.7 ± 4.6	22.5 ± 5.3	18.9 ± 4.2	37.8 ± 3.5	43.2 ± 3.8	37.5 ± 3.6	28.1 ± 3.3	32.9 ± 3.0	29.6 ± 3.3
**5. OPL**	23.0 ± 3.4	20.7 ± 4.9	20.3 ± 5.3	29.6 ± 3.8	28.0 ± 5.0	30.0 ± 5.1	26.2 ± 2.6	24.1 ± 3.0	28.4 ± 4.2
**6. OPL-HFL ~BMEIS**	120.8 ± 10.2	122.3 ± 9.2	116.5 ± 10.7	95.5 ± 8.8	96.9 ± 9.2	90.3 ± 8.8	79.7 ± 10.1	79.4 ± 6.4	71.6 ± 6.8
**7. IS/OS**	13.9 ± 0.9	11.7 ± 0.6	14.8 ± 2.4	12.6 ± 0.6	10.3 ± 0.4	13.7 ± 1.7	12.5 ± 1.2	10.2 ± 0.8	13.0 ± 1.7
**8. IS/OSJ ~IB_OPR**	17.0 ± 1.8	19.7 ± 2.2	17.3 ± 3.0	11.7 ± 1.4	14.0 ± 2.9	12.6 ± 2.4	10.3 ± 2.1	15.5 ± 4.3	15.8 ± 4.4
**9. OPR**	20.1 ± 2.6	20.7 ± 3.0	20.6 ± 3.8	19.8 ± 2.2	21.3 ± 3.3	20.7 ± 3.4	18.5 ± 2.3	17.8 ± 4.8	15.2 ± 3.8
**10. RPE**	18.6 ± 2.1	15.5 ± 0.5	15.6 ± 0.2	18.6 ± 1.7	15.5 ± 0.4	15.5 ± 0.2	18.6 ± 1.6	15.3 ± 0.5	15.5 ± 0.4

**Table 2:** Total retinal thickness measurements from different segmentation methods. Thickness values (mean ± SD; μm) for the three ETDRS regions of images acquired at session 1.

		Fovea	Inner ring	Outer ring
**Iowa Reference Algorithms**	1040nm	270.9 ± *16*.*8*	335.2 ± *17*.*4*	294.0 ± *20*.*6*
	Topcon	281.5 ± *16*.*9*	342.8 ± *18*.*0*	295.2 ± *21*.*8*
	Zeiss	280.8 ± *17*.*5*	347.0 ± *18*.*0*	302.0 ± *21*.*4*
**On-board segmentation**	Topcon	244.6 ± *17*.*4*	306.0 ± *17*.*6*	260.9 ± *21*.*4*
	Zeiss	263.1 ± *17*.*4*	325.4 ± *18*.*1*	281.2 ± *21*.*4*
Iowa equivalent retinal thickness*	Topcon	242.8 ± *22*.*9*	304.4 ± *19*.*3*	258.0 ± *21*.*6*
	Zeiss	265.4 ± *18*.*6*	331.6 ± *18*.*7*	286.8 ± *21*.*6*

*Equivalent values from the Iowa Reference Algorithms (Topcon, ILM to inner boundary of OPR; Zeiss, ILM to inner boundary of RPE) are quoted for comparison to the commercial on-board software segmentation.

An inter-device comparison of AEL-corrected scaled retinal thickness measurements produced by the Iowa Reference Algorithms is shown in Table 3. One significant outlier was removed from the 1040nm-Topcon comparison (foveal subfield only). Images from the Topcon instrument yielded significantly higher values for retinal thickness than the long-wavelength system (Friedman test, P<0.05), but significantly lower values than the Zeiss OCT (with the exception of the foveal subfield). This bias between the Zeiss and long-wavelength OCT images was similar across the three ETDRS subfields. However, the bias between the Topcon and long-wavelength images decreases in magnitude with eccentricity (largest in the foveal subfield and smallest in the outer ring).

**Table 3:** Agreement of total retinal thickness between OCT devices. Mean difference (bias; μm) and 95% limits of agreement (μm) for mean retinal thickness at session 1 produced by the Iowa Reference Algorithms, using AEL-dependent scaling, for each pairing of OCT instruments.

		Fovea	Inner ring	Outer ring
**1040nm-Topcon**	Mean difference	-14.18	-7.05	0.80
	*Limits of agreement*	*-20*.*05 to -8*.*30*	*-14*.*65 to 0*.*55*	*-6*.*95 to 8*.*55*
	Friedman test P	0.000*	0.000*	0.317
**1040nm-Zeiss**	Mean difference	-13.89	-12.46	-6.62
	*Limits of agreement*	*-21*.*62 to -6*.*16*	*-18*.*91 to -6*.*00*	*-13*.*53 to 0*.*30*
	Friedman test P	0.000*	0.000*	0.000*
**Topcon-Zeiss**	Mean difference	-0.21	-5.41	-7.42
	*Limits of agreement*	*-4*.*18 to 3*.*75*	*-8*.*67 to -2*.*15*	*-10*.*66 to -4*.*17*
	Friedman test P	0.841	0.000*	0.000*

*significant at 0.05 level.

**Fig 2:**
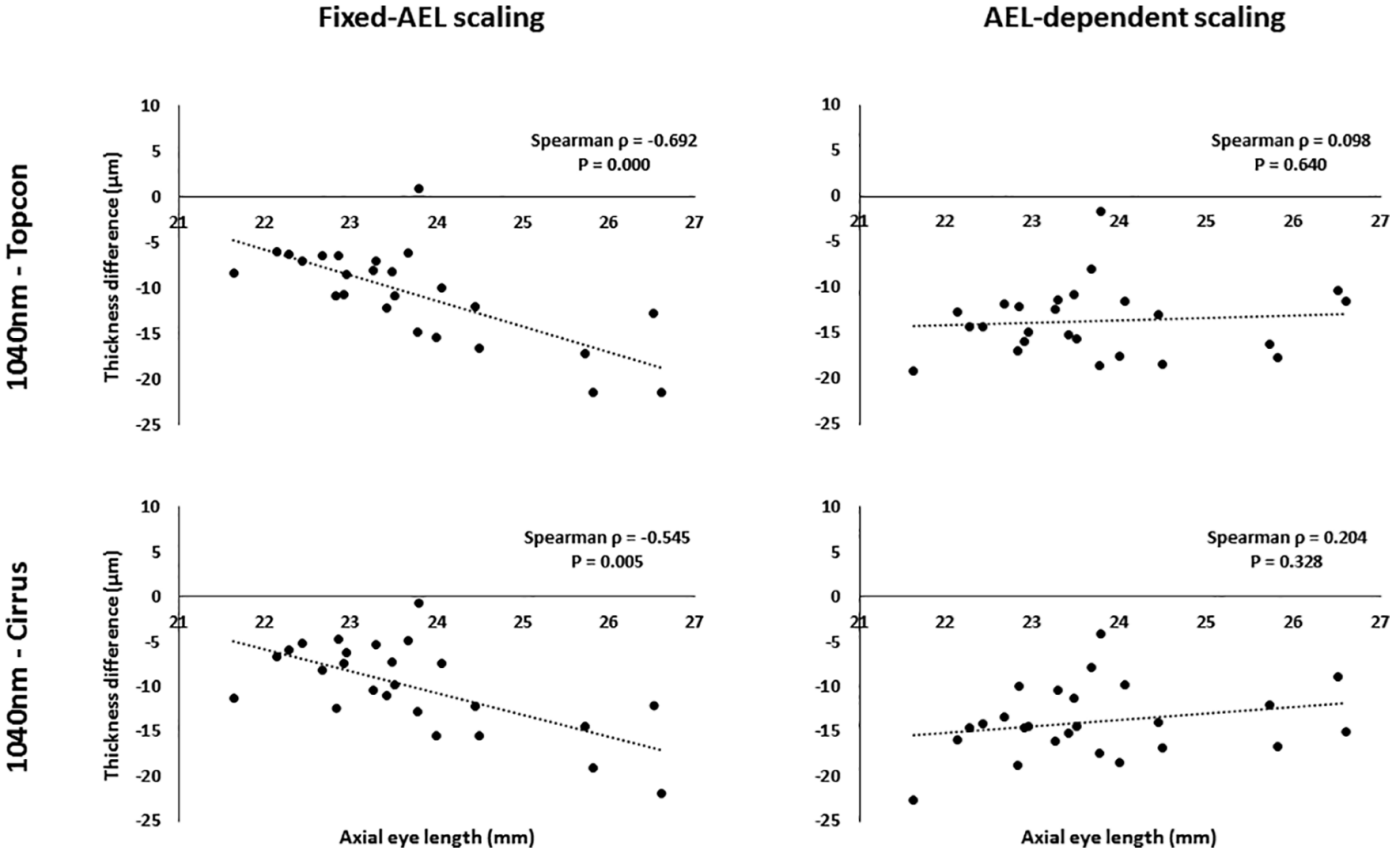
Correlation of retinal thickness differences against AEL. Difference in retinal thickness measurements (μm) versus axial eye length (mm) for the foveal subfield, as produced by the Iowa Reference Algorithms using two different transverse scaling methods.

Discrepancies in fixed-AEL scaled retinal thickness measurements between the commercial and long-wavelength devices showed a strong correlation with AEL, for the commercial instrument data, in both the foveal subfield and the outer ring (Spearman rank ρ>0.50, P<0.05 in all cases), although only moderate to weak for the inner ring (Spearman ρ<0.50). A weak correlation was also seen across all subfields when comparing Topcon with Zeiss data. In all cases, the correlation was reduced and/or became not significant when the AEL-dependent scaling was used in the comparisons between data from any of the three sources (Fig 2).

### Inter-Session Repeatability

**Fig 3:**
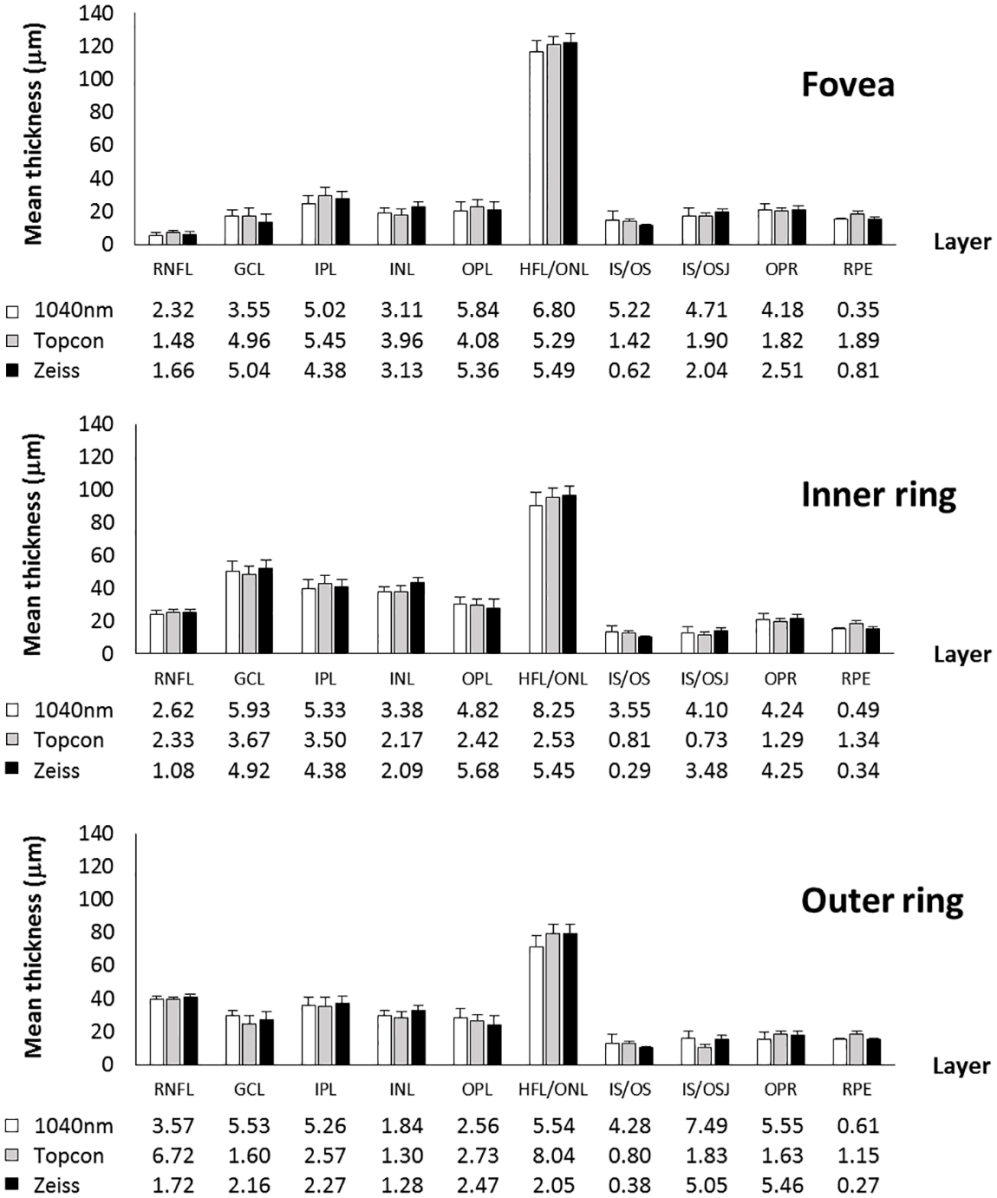
Intra-retinal layer thickness and CoR. Mean thickness of 10 intra-retinal layers segmented by the Iowa Reference Algorithms at session 1, on images from all three OCT devices. Error bars and table values represent inter-session CoR (μm) for each layer.

Intra-retinal layer thickness values produced by the Iowa software were compared between session 1 and session 2. For these comparisons, AEL-corrected scaled data was used. Differences outside 3 SD of the mean difference (Grubbs’ test) were considered failings in segmentation and were removed from further analysis (n = 26 of 2250 comparisons). Of these, 46%, 42% and 12% were attributed to images from the long-wavelength, Topcon and Zeiss instruments respectively. In general, CoR was similar across images from all devices, and across the three ETDRS subfields (Fig 3).

For layer thickness averaged across the entire ETDRS grid, the layers with best repeatability (as a percentage of the mean) were layer 10 (RPE; 1.2%), layer 4 (INL; 6.2%), and layer 10 (RPE; 4.1%) for the Zeiss, Topcon and long-wavelength devices respectively. The layers with poorest repeatability were layer 8 (IS/OSJ ~ IB_RPE; 22.5%), layer 1 (RNFL; 19.1%), and layer 8 (IS/OSJ ~ IB_RPE; 36.9%) respectively.

Total retinal thickness values were also compared between session 1 and session 2. In all cases, the mean bias was between ±1 μm, and there was no significant difference between session 1 and session 2 for any device or ETDRS subfield (P>0.05 in all cases). Fixed-AEL scaling was used to allow comparison to the commercial segmentation. One significant outlier was removed from the long-wavelength data (all subfields), as identified by Grubbs’ test. For the Iowa Reference Algorithms, the CoR for the foveal subfields was 4.76μm and 3.43μm for Topcon and Zeiss OCT images respectively. The CoR of both instruments was lower than that of the long-wavelength OCT, which had a CoR of 5.98μm. In all cases the CoR was higher for the foveal subfield than the inner and outer rings (Table 4).

**Table 4:** Inter-session repeatability of the Iowa Reference Algorithms. Coefficients of repeatability (μm; and percentage) of mean retinal thickness produced by the Iowa Reference Algorithms at session 1 and session 2.

		Fovea	Inner ring	Outer ring
**1040nm**	Full thickness	5.98 (*2*.*2%*)	5.86 (*1*.*7%*)	5.48 (*1*.*9%*)
**Topcon**	Full thickness	4.76 (*1*.*7%)*	3.99 (*1*.*2%*)	4.10 (*1*.*4%*)
	Equivalent thickness*	3.90 (*1*.*6%*)	3.48 (*1*.*1%*)	3.78 (*1*.*5%*)
**Zeiss**	Full thickness	3.43 (*1*.*2%*)	2.92 (*0*.*8%*)	3.19 (*1*.*1%*)
	Equivalent thickness*	3.37 (*1*.*3%*)	2.95 (*0*.*9%*)	3.14 (*1*.*1%*)

*Equivalent values from the Iowa Reference Algorithms (Topcon, ILM to inner boundary of OPR; Zeiss, ILM to inner boundary of RPE) are quoted for comparison to the commercial on-board software segmentation.

For the commercial segmentation, no AEL-correction was made in the scaling. Two significant outliers were removed from the Topcon segmentation data (foveal and inner ring subfields only). The CoR of the foveal subfield was 7.63μm and 4.63μm for the Topcon and Zeiss OCT respectively. Again, the CoR was higher for the foveal subfield than the inner and outer rings for the Topcon OCT images, but was similar across all subfields for the Zeiss OCT images (Table 5).

**Table 5:** Inter-session repeatability of the on-board software. Coefficients of repeatability (μm; and percentage) of mean retinal thickness produced by the on-board analysis software of the commercial instruments at session 1 and session 2.

	Fovea	Inner ring	Outer ring
**Topcon**	7.63 (*3*.*1%*)	4.68 (*1*.*5%*)	5.68 (*2*.*2%*)
**Zeiss**	4.63 (*1*.*8%*)	5.06 (*1*.*6%*)	5.04 (*1*.*8%*)

**Fig 4:**
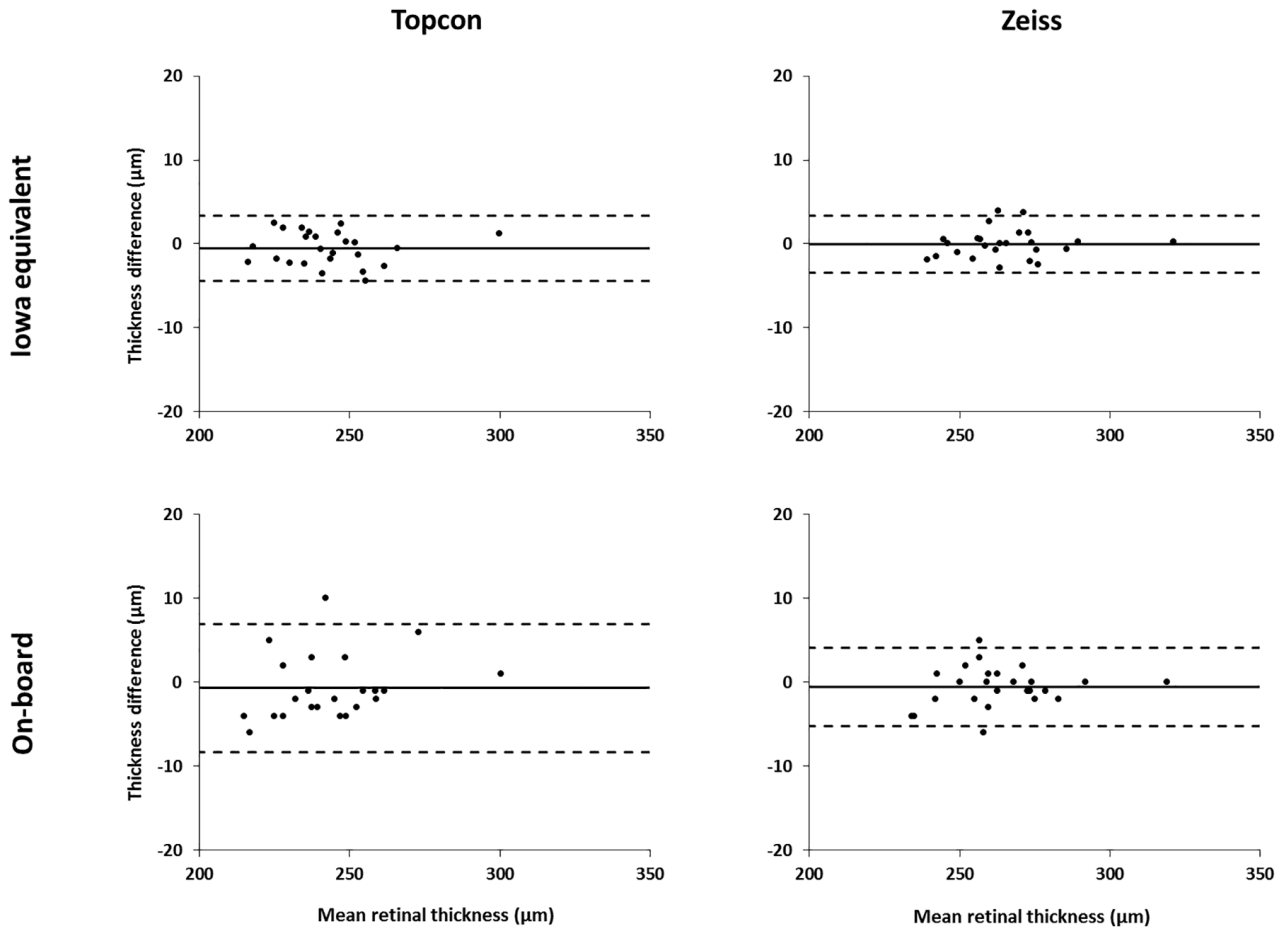
Bland-Altman plots showing inter-session repeatability. Total retinal thickness difference (μm) against mean (μm) for inter-session repeatability of the foveal subfield for both segmentation methods. 95% limits of agreement shown by dashed lines. Note that two significant outliers were removed from the Topcon on-board segmentation data.

**Fig 5:**
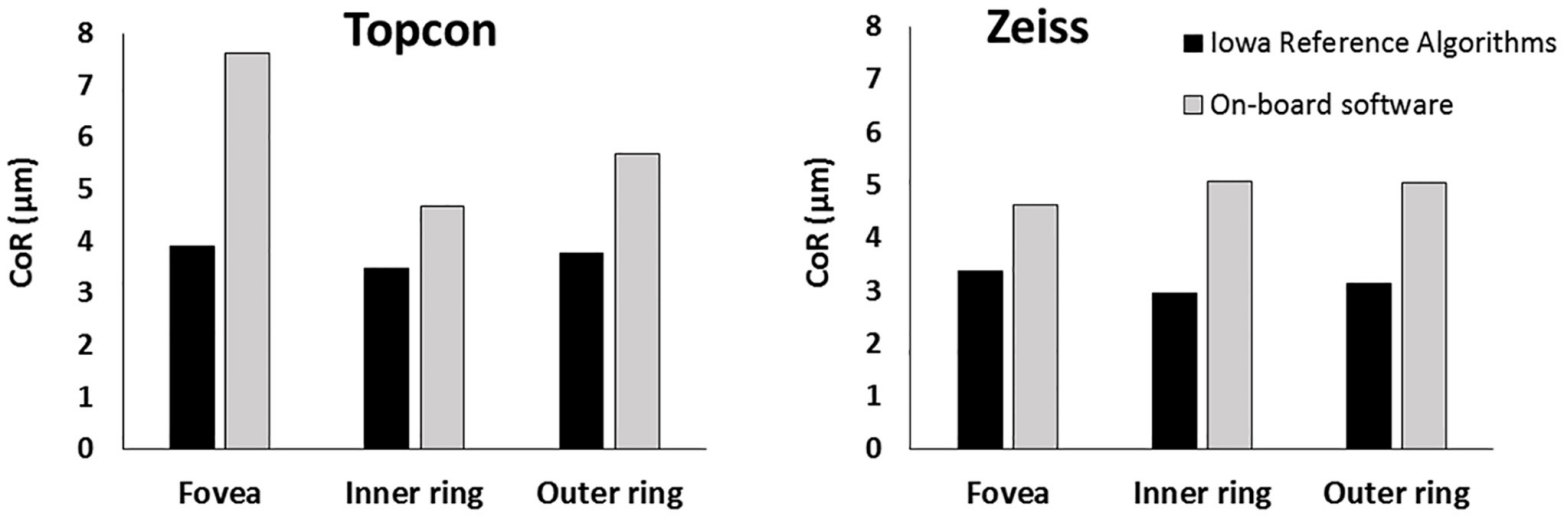
Comparison of inter-session repeatability for the two segmentation methods. Coefficients of repeatability for segmentation by the Iowa Reference Algorithms and on-board software are shown for the Topcon (left) and Zeiss (right) systems. The ‘equivalent retinal thickness’ values from the Iowa Reference Algorithms were used in this analysis.

To allow for comparison between segmentation methods, the Iowa Reference Algorithm ‘equivalent retinal thicknesses’ were used (Table 4; Fig 4). The on-board software of both commercial instruments was less repeatable than the Iowa Reference Algorithms, for all three subfields (Fig 5).

### Segmentation Software Agreement

A comparison of retinal thickness measurements produced by the Iowa Reference Algorithms and the commercial segmentation software for the two commercial instruments is shown in Table 6. Again, the thickness values were fixed-AEL scaled to allow comparison between algorithms. One significant outlier was removed from the analysis of the Topcon data (foveal subfield only). The commercial segmentation software yielded similar to or marginally higher retinal thickness values than the Iowa Reference Algorithms for the Topcon OCT images. These differences were significant only in the outer ring (Friedman test, P<0.05). The commercial software yielded significantly lower values than the Iowa Reference Algorithms for the Zeiss OCT images in all three subfields (Friedman test, P<0.05).

**Table 6:** Agreement of total retinal thickness between segmentation methods. Mean difference (bias; μm) and 95% limits of agreement (μm) for mean retinal thickness at session 1 produced by the Iowa Reference Algorithms and the commercial on-board equivalent.

		Fovea	Inner ring	Outer ring
**Topcon**	Mean difference	0.11	-1.65	-2.91
	*Limits of agreement*	*-13*.*05 to 13*.*28*	*-10*.*96 to 7*.*66*	*-12*.*97 to 7*.*15*
	Friedman test P	0.414	0.549	0.009*
**Zeiss**	Mean difference	2.00	6.07	5.73
	*Limits of agreement*	*-5*.*76 to 9*.*75*	*-5*.*95 to 17*.*82*	*-6*.*03 to 16*.*42*
	Friedman test P	0.028*	0.003*	0.009*

*significant at 0.05 level

## Discussion

The Iowa Reference Algorithms produced automated segmentation of 10 intra-retinal layers on all images from all three devices. We have demonstrated a substantial impact of AEL-corrected lateral scaling on mean intra-retinal layer thickness values. If an AEL-dependent lateral scaling is not used, the ETDRS grid (defined in mm units at the retinal surface) will overlay a smaller or larger area of retina, depending on the AEL. The ETDRS subfield sizes could vary by up to 30% assuming a normal AEL range of 20-28mm, which is a little larger than for the cohort of this present study. Appropriate scaling to account for AEL is therefore an important consideration for any quantitative retinal thickness analysis, particularly in cross-sectional applications.

The Iowa Reference Algorithms demonstrated good agreement between the Topcon and Zeiss instruments (as shown previously; [33]), and with the long-wavelength device. However, the Iowa Reference Algorithms produced significantly higher retinal thickness values on images from both commercial systems than the long-wavelength system, across all subfields, even when the AEL-dependent scaling was used. Refractive index assumptions for retinal tissues used by each instrument could result in a small difference in axial scaling. Knowing the assumptions made for each device, we expect a difference in absolute thickness values between instruments of between 1.4% and 2.9%, with largest thickness values from the Zeiss images (n = 1.36), then Topcon images (n = 1.38) and finally the long-wavelength OCT (n = 1.4). In general, this is consistent with our findings (Table 2).

Discrepancies in retinal thickness measurement between images from the long-wavelength and commercial systems were significantly correlated with AEL for the foveal and outer ring subfields, when using the fixed-AEL scaling. When the AEL-dependent scaling was used throughout this analysis, all these correlations were reduced and were no longer significant (Fig 2). This effect is consistent with the expected transverse magnification error that arises when fixed-AEL scaling is used for images from either of the commercial instruments. This confirms that the on-board software of the Zeiss and Topcon instruments used in this study do not make a scaling correction for AEL. The Iowa Reference Algorithms allow for direct input of transverse scaling factors prior to image segmentation which should be used, where possible, to reduce these demonstrated magnification errors.

Inter-session repeatability of the Iowa Reference Algorithms was assessed for each of the 10 intra-retinal layers on images from each device. Although the CoR was similar across the three devices and three ETDRS subfields, there were far fewer significant outliers excluded from the Zeiss data by Grubbs’ test. Had these outliers been included, inter-session repeatability would have been poorer for the other two devices. Overall, RPE thickness was the most repeatable measure, particularly for the Zeiss and long-wavelength OCT (CoR 0.5μm for both). As a change in thickness of this layer greater than this value would indicate change outside the normal variation, these measures represent a useful clinical biomarker for diseases that affect the RPE, such as AMD [34].

The least repeatable measures were the inner retinal layers (1–5) in the foveal subfield (Fig 3). This is expected, since these layers are thin in this region. Additionally, some layers are more difficult to distinguish due to near iso-reflectivity, for example GCL from IPL, and HFL from OPL. The HFL commonly fluctuates in appearance between acquisitions, dependent on angle of incidence, which would also likely limit the repeatability of segmentation of this layer [35]. The inner retinal layers were generally more repeatable in the outer ring across all devices. However, the outer ring has the lowest RNFL repeatability across all devices, possibly attributed to the thickness variability due to nasal/temporal asymmetry at this eccentricity. However, if analyzed in an appropriate manner (by quadrant, for instance), this may have applications in a number of conditions [36–39].

Total retinal thickness in the foveal subfield was less repeatable than the inner and outer rings, which is also likely attributable to retinal anatomy (greater thickness variability in the foveal subfield). All repeated measures using the Iowa Reference Algorithms on the Topcon and Zeiss OCT images were within 1.7% and 1.3% of mean retinal thickness respectively for all subfields (Table 4). The long-wavelength OCT images had a marginally higher CoR (within 2.2% for all subfields, with one outlier removed). This non-commercial device was designed for optimal imaging of the deeper structures, including the choroid, due to reduced scatter at this wavelength [25]. However, differences in reflective properties of the layer boundaries are likely to occur at this longer wavelength, which the Iowa Reference Algorithms may not be optimized to detect. Since the main function of the long-wavelength OCT is to image the deeper retinal structures, the acquisition protocol used to collect these images was similar to enhanced depth imaging [40], with the choroid intentionally positioned closer to the zero delay line. This may have resulted in reduced signal from the more superficial retinal layers, and may explain the reduced repeatability compared to the commercial devices.

The Iowa Reference Algorithms measurements were more repeatable than the equivalent commercial segmentation software assessed. The inter-session repeatability analysis of the Topcon commercial segmentation software revealed two clear outliers. Both images were of poorer quality than the rest of the sample (image quality <25 in the Topcon-generated report) which led to marked errors in ILM boundary position in the foveal subfield during segmentation. Removing these outliers from this subfield, the CoR was reduced from 21.56μm to 7.63μm, which is more comparable to the other subfields and to the Zeiss commercial software. It should be noted that segmentation of these same two images using the Iowa Reference Algorithms produced no marked errors in boundary placement in any subfield.

Since the majority of these CoR values are nearing the resolution limit of all three instruments (~5μm axially), this small inter-session thickness variation is unlikely to be clinically meaningful. However, since many thickness values are averaged over each region and/or over the entire cohort in this study, significant results of boundary positions can be obtained to a higher precision than the optical resolution of the instrument. The axial sampling resolution (3.5μm for the Topcon, and ~2μm for the Zeiss and long-wavelength systems) is another potential limiting factor but averaging over many thickness measurements can again, in theory, provide even higher resolution results limited ultimately by the signal-to-noise ratio of the devices and the repeatability of the scanning geometry.

Our inter-session findings for the Zeiss instrument are comparable to, if not slightly more repeatable than, previous findings [11,41]. Kotera and colleagues [42] reported slightly higher repeatability than our findings for the Topcon segmentation software (total retinal thickness CoR of 3.10μm and 2.01μm for the inner ring and outer ring respectively, in comparison to our values of 5.99μm and 5.68μm). However, their assessment was an intra- rather than inter-session repeatability, and did not include the ETDRS foveal subfield since their main outcome measure was retinal nerve fiber layer (RNFL) thickness in glaucoma. It is worth noting that we would expect the inter-session repeatability to be poorer in eyes with macular disease due to the nature of the pathological retinal features [43,44]. In some cases, the segmented boundaries may require manual repositioning to ensure accurate quantification. Measures in healthy participants were the focus of the present study, to evaluate the repeatability of the algorithm under ideal conditions.

Good agreement was shown between the Iowa Reference Algorithms and the commercial software, when equivalent segmentation boundaries were used (as shown in Table 6). There was slight bias in both cases, with the Topcon segmentation producing slightly higher values (Friedman test, P<0.05 in the outer ring only), and the Zeiss segmentation producing lower values than the Iowa Reference Algorithms (Friedman test, P<0.05 in all subfields). However, these discrepancies accounted for less than 2% of total retinal thickness in all subfields, and are unlikely to be significant in clinical applications. This is consistent with previous comparative studies using the Iowa Reference Algorithms and Cirrus and Spectralis OCT commercial software [17,45]. To our knowledge, no previous comparison has been made using the Topcon 3D-OCT.

This study had a relatively small sample size (n = 25) for an inter-session repeatability study and a relatively narrow distribution of AEL. Only participants without retinal disease were included in this study, therefore conclusions can only be applied to this group. Further study is necessary to assess the repeatability of the segmentation software in ocular disease. Lastly, the high repeatability of the intra-retinal layer segmentation by the Iowa Reference Algorithms is promising, although further analysis is required to compare this with the available on-board software.

In conclusion, we have confirmed that lateral magnification errors affect the consistency and reliability of generated thickness values, and are therefore an important consideration in quantitative OCT retinal layer analysis. Nevertheless, the Iowa Reference Algorithms provide repeatable automated retinal thickness measurements, which outperform the commercial segmentation software, and allow a convenient mechanism to apply an accurate AEL-dependent lateral scaling to images from any OCT device. In these respects, this algorithm is viable for clinical and research applications, for eyes without ocular disease imaged using the three OCT devices in this study.
